# One Health genomics reveals niche-specific lineage replacement in *Salmonella* Enteritidis

**DOI:** 10.1093/nsr/nwag275

**Published:** 2026-05-12

**Authors:** Haiyang Zhou, Linlin Huang, Jiansen Gong, Chenghao Jia, Qianzhe Cao, Zubin Wang, Yan Song, Jiaqi Chen, Aizhong Chen, Yi Zhang, Abdelaziz Ed-Dra, Yan Li, Guoping Zhao, Min Yue

**Affiliations:** Department of Veterinary Medicine, Zhejiang University College of Animal Sciences, Hangzhou 310058, China; Key Laboratory of Systems Health Science of Zhejiang Province, School of Life Science, Hangzhou Institute for Advanced Study, University of Chinese Academy of Sciences, Hangzhou 310024, China; Department of Veterinary Medicine, Zhejiang University College of Animal Sciences, Hangzhou 310058, China; Key Laboratory for Poultry Genetics and Breeding of Jiangsu Province, Jiangsu Institute of Poultry Sciences, Yangzhou 225125, China; Department of Veterinary Medicine, Zhejiang University College of Animal Sciences, Hangzhou 310058, China; Key Laboratory of Systems Health Science of Zhejiang Province, School of Life Science, Hangzhou Institute for Advanced Study, University of Chinese Academy of Sciences, Hangzhou 310024, China; Department of Veterinary Medicine, Zhejiang University College of Animal Sciences, Hangzhou 310058, China; Key Laboratory of Systems Health Science of Zhejiang Province, School of Life Science, Hangzhou Institute for Advanced Study, University of Chinese Academy of Sciences, Hangzhou 310024, China; Department of Veterinary Medicine, Zhejiang University College of Animal Sciences, Hangzhou 310058, China; Department of Veterinary Medicine, Zhejiang University College of Animal Sciences, Hangzhou 310058, China; Key Laboratory of Systems Health Science of Zhejiang Province, School of Life Science, Hangzhou Institute for Advanced Study, University of Chinese Academy of Sciences, Hangzhou 310024, China; Key Laboratory of Systems Health Science of Zhejiang Province, School of Life Science, Hangzhou Institute for Advanced Study, University of Chinese Academy of Sciences, Hangzhou 310024, China; Laboratory of Engineering and Applied Technologies, Higher School of Technology, M’ghila Campus, Sultan Moulay Slimane University, Beni Mellal 23000, Morocco; Department of Veterinary Medicine, Zhejiang University College of Animal Sciences, Hangzhou 310058, China; Key Laboratory of Systems Health Science of Zhejiang Province, School of Life Science, Hangzhou Institute for Advanced Study, University of Chinese Academy of Sciences, Hangzhou 310024, China; CAS Key Laboratory of Synthetic Biology, Institute of Plant Physiology and Ecology, Shanghai Institutes for Biological Sciences, Chinese Academy of Sciences, Shanghai 200031, China; Department of Microbiology and Microbial Engineering, School of Life Sciences, Fudan University, Shanghai 200433, China; Department of Veterinary Medicine, Zhejiang University College of Animal Sciences, Hangzhou 310058, China; Key Laboratory of Systems Health Science of Zhejiang Province, School of Life Science, Hangzhou Institute for Advanced Study, University of Chinese Academy of Sciences, Hangzhou 310024, China; State Key Laboratory for Diagnosis and Treatment of Infectious Diseases, National Clinical Research Center for Infectious Diseases, National Medical Center for Infectious Diseases, The First Affiliated Hospital, College of Medicine, Zhejiang University, Hangzhou 310003, China

**Keywords:** *Salmonella* Enteritidis, genomics, antimicrobial resistance, lineage replacement, population evolution

## Abstract

*Salmonella enterica* serovar Enteritidis (S. Enteritidis) is a broad-host-range pathogen posing global challenges to livestock production and public health. However, nation-scale evolutionary dynamics of Enteritidis remain unexplored, considering emerging evidence of human-adaptive traits. Here, we employed a transdisciplinary One Health approach to compile and analyze the largest genomic dataset of *S*. Enteritidis in China, integrating isolates across human, animal, food, and environmental sectors. Our analyses reveal an alarming escalation in antimicrobial resistance (AMR), with multidrug resistance rates tripling over the two decades. This surge in AMR coincides with the divergent evolution of three major Chinese Mainland lineages: Global-b1, Global-b2, and Global-c. Crucially, we demonstrate that the Global-c lineage has achieved proportional dominance through a process of resistance-driven lineage replacement. This success is fueled by mobile genetic elements and supported by enhanced environmental stress tolerance and metabolic plasticity. Additional SNP-distance mapping uncovered evidence of human-centric dissemination. Collectively, we elucidate the complex, localized adaptation and competitive dynamics of *S*. Enteritidis lineages.

## INTRODUCTION

*Salmonella enterica* serovar Enteritidis (*S*. Enteritidis) is a leading cause of foodborne outbreaks and invasive infections globally [1,2]. As a broad-host-range pathogen, it poses a persistent threat to livestock—particularly poultry—and human health [3,4]. While *S*. Enteritidis is frequently implicated in large-scale foodborne gastrointestinal outbreaks in high-income countries [5], it is a primary driver of invasive non-typhoidal *Salmonella* (iNTS) disease in low- and middle-income regions [1,6]. In Africa, the continent most burdened by iNTS, *S*. Enteritidis represents a critical infection risk, second only to *S*. Typhimurium ST313 [7,8]. Evidence suggests that these serovars are undergoing parallel evolution, characterized by progressive adaptation to the human host [9–11].

However, a recent study indicates that, outside Africa, such as in China, *S*. Enteritidis has diverged into increasingly antimicrobial-resistant (AMR) and human-adapted lineages that follow evolutionary trajectories distinct from African strains [12,13]. These regional investigations underscore a pronounced geographic dependency in *S*. Enteritidis evolution, where specific ecological niches give rise to region-specific lineages [14]. For instance, while the Central/Eastern and West African clades remain largely endemic to the African continent [1], the Global-c clade is predominantly identified in China [12]. Similarly, geographic restriction is observed in MGT5-STs within the United States, the United Kingdom

[15], and Australia [16]. Given that host adaptation is a central force driving population diversity [17], elucidating the regional pathoadaptive parameters is imperative for assessing transmission potential and public health risk [18–21].

The efficacy of antimicrobials, the cornerstone of salmonellosis clinical management, is increasingly compromised by the global escalation of AMR. The World Health Organization has designated non-typhoidal *Salmonella* as a ‘top-ranked’ Bacterial Priority Pathogen [22], reflecting the urgency of the crisis. China, a global hotspot for antimicrobial consumption, is experiencing a significant rise in AMR prevalence. The country’s vast population and intensive agricultural sector provide a substantial selective environment for resistant strains [23], with Chinese *Salmonella* isolates frequently exhibiting higher resistance levels than those from other geographic regions [24–26]. The emergence of strains resistant to front-line antimicrobials further challenges conventional control measures. Due to its broad host range and presence across various interfaces, *S*. Enteritidis serves as an ideal One Health sentinel, facilitating the integrated assessment of resistance profiles across human, animal, and environmental sectors to guide targeted interventions.

Building on our previous identification of a distinctive multidrug-resistant (resistant to three or more antimicrobial classes, MDR) Chinese lineage with potential for human-to-human transmission [12], there remains a critical knowledge gap regarding the One Health dynamics of *S*. Enteritidis in China. Considering the serovar’s strong geographic dependence and its ubiquity across diverse hosts, a comprehensive, multi-sectoral understanding is required. To address this, we employed a large-scale transdisciplinary approach, sequencing and analyzing 2415 *S*. Enteritidis isolates from humans, animals, food, and the environment. This integrated analysis of nation-scale datasets allows us to map the evolutionary landscape of *S*. Enteritidis in China and propose data-driven mitigation strategies to inform rational public health policy.

## RESULTS

### Spatial-temporal shifts and source dynamics of *S*. Enteritidis in China

Following initial data collection and metadata curation (Fig. S1a), we analyzed a comprehensive dataset of 2415 *S*. Enteritidis genome sequences from China, integrating public databases with routine surveillance data. Spatiotemporal analysis revealed a shifting geographic distribution: during 2004–13, the isolates were mainly reported in eastern regions, particularly Shandong and Taiwan. In the most recent decade (2014–23), *S*. Enteritidis has been documented across most provinces nationwide, with Shandong and Zhejiang emerging as the primary sources (Table S1).

**Figure 1.**
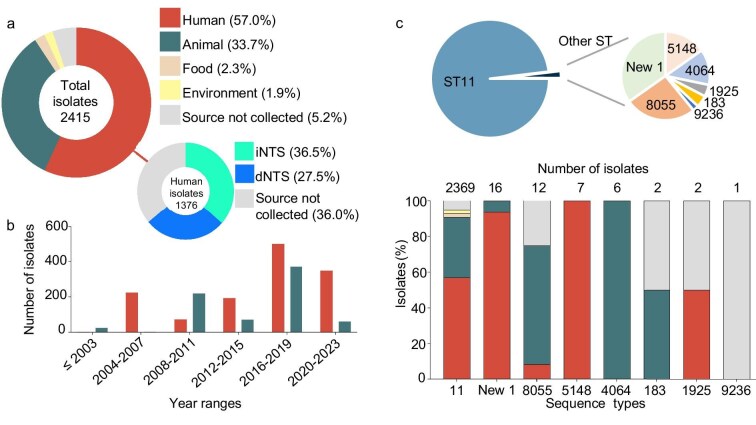
Source composition, temporal distribution, and sequence type (ST) analysis of collected *Salmonella* isolates. (a) Composition of sample sources, predominantly from humans and animals, with human-derived samples further classified into invasive non-typhoidal *Salmonella* (iNTS) and diarrheal non-typhoidal *Salmonella* (dNTS). (b) Temporal trends in the number of *Salmonella* isolates from animal and human sources. (c) The upper pie chart illustrates the proportion of different sequence types (STs) among all strains, while the lower bar chart shows the host source distribution (human, animal, food, environment) within each ST type. The number of isolates in each ST type is labeled.

Of the 2415 isolates, 57.0% (1376/2415) were of human origin. Notably, 36.5% (502/1376) of these were confirmed as iNTS (Fig. 1a), recovered primarily from blood (91.83%, 461/502) and pus (3.59%, 18/502) (Fig. S1b). The human-derived isolates included 261 male and 179 female patients; pediatric cases (<18 years old) accounted for ∼40% of the characterized infections (Fig. S1c). Beyond human clinical samples, 33.7% (814/2415) of the isolates were obtained from animals—predominantly food-producing livestock. A smaller fraction originated from food sources (2.3%, 55/2415), primarily poultry meat (specifically chicken), while 45 environmental isolates were recovered, mainly from agricultural settings (Fig. S1d).

The nationwide isolation of *S*. Enteritidis increased steadily over time, peaking between 2016 and 2019. A slight decline in detections was observed after 2020, likely attributable to reporting lags or reduced surveillance capacity during the COVID-19 pandemic [27]. Intriguingly, the temporal distribution of animal‑derived versus human‑derived isolates exhibited a distinct bimodal pattern. This alternating, out‑of‑phase surge suggests that the pathogen burden in animal reservoirs and human cases oscillated sequentially rather than rising in synchrony (Fig. 1b).

Chinese *S*. Enteritidis were classified into eight different sequence types (STs). ST11 was overwhelmingly dominant, accounting for 98.10% (2369/2415) of all strains. We also identified a novel ST, STnew1 [*aroC*(5), *dnaN*(2), *hemD*(3), *hisD*(1697), *purE*(6), *sucA*(6), *thrA*(11)]. The STs exhibited clear differences in host range: while ST11 demonstrated high plasticity—circulating across human, animal, food, and environmental interfaces—other STs appeared more host-restricted. Specifically, ST5148, ST1925, and STnew1 were primarily associated with human infection, whereas ST183, ST4064, and ST8055 were predominantly isolated from animal sources (Fig. 1c).

### Widespread and escalating AMR among *S*. Enteritidis isolates

**Figure 2.**
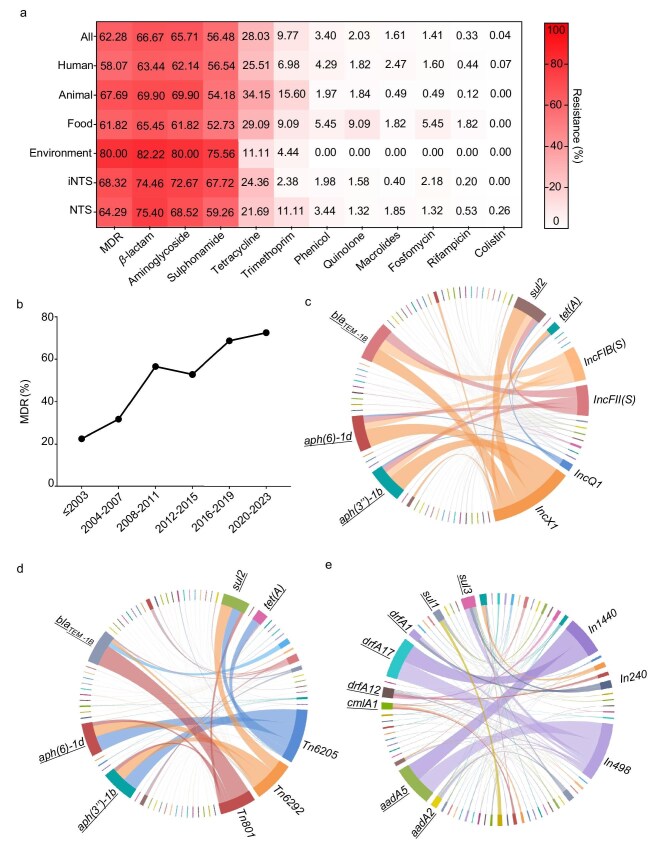
Antimicrobial resistance profiles and genetic associations. (a) Percentage of resistance genotypes across different sample sources. Isolates carrying resistance genes for specific antimicrobials are considered resistant; the gene *aac(6')-Iaa* was excluded from the analysis. (b) Proportion of multidrug-resistant (MDR) strains across different periods. (c) Co-localization of plasmids and resistance genes, illustrating their associations. ARGs are underlined to better distinguish them from plasmids. (d) Co-localization of transposons and resistance genes, showing their distribution and relationships. ARGs are underlined to better distinguish them from integrons. (e) Co-localization of integrons and resistance genes, highlighting their interconnectedness. ARGs are underlined to better distinguish them from transposons.

Utilizing detected antimicrobial resistance genes (ARGs), we assigned antimicrobial‑resistance genotypes to all isolates. *β*‑lactam, aminoglycoside, and sulphonamide resistance determinants were the most prevalent, with over 50% of isolates carrying the corresponding ARGs. Additionally, 28% of the population harbored tetracycline resistance genes, while ∼9% carried trimethoprim resistance genes. Notably, source-specific resistance patterns emerged: environmental *Salmonella* exhibited the highest overall resistance (80% MDR rate) despite a relatively low tetracycline‑resistance rate (11.11%). In contrast, animal-derived strains displayed pronounced resistance to tetracycline and trimethoprim, while food‑borne isolates were characterized by elevated resistance to quinolones and fosfomycin (Fig. 2a). Overall, 62% of strains were multidrug‑resistant, and the MDR prevalence increased steadily over the study period (Fig. 2b).

To elucidate the genetic basis of these ARGs, we mapped the ARGs to mobile genetic elements (MGEs). We identified that plasmids *IncX1, IncFIB(S)* and *IncFII(S)*, along with transposons *Tn6205, Tn6292* and *Tn801*, served as major vehicles for aminoglycoside and *β*‑lactam resistant genes—*aph(6)‑Id, aph(3'')‑Ib* and *bla*_TEM‑1b_—and were also strongly associated with *sul(2)* and *tet(A)*. Furthermore, integrons were identified as the primary reservoirs for trimethoprim resistance genes and *aadA5* (Fig. 2c–e).

### MGEs-mediated ARGs enrichment associates with Global-c clade expansion

**Figure 3.**
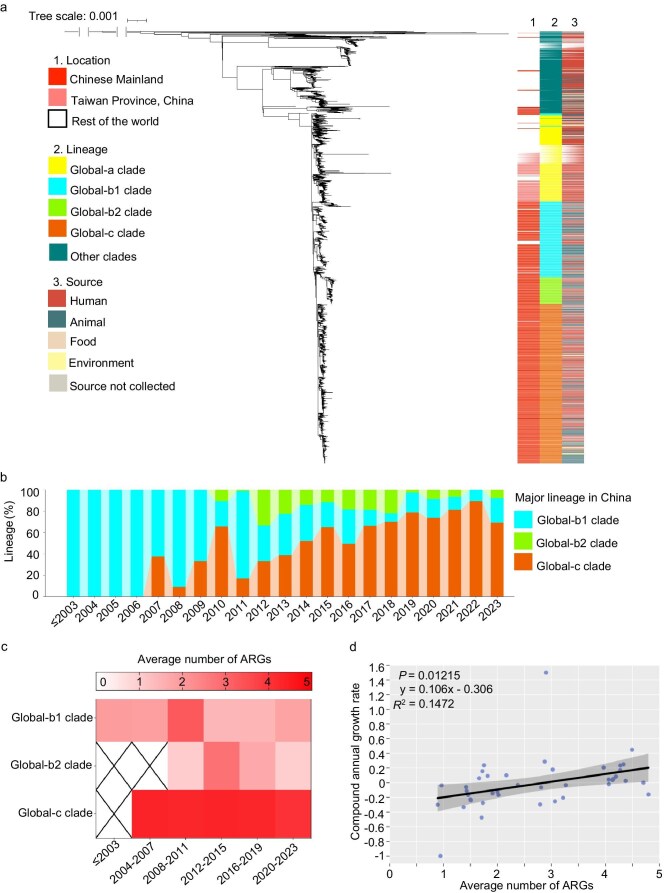
Phylogenetic analysis, lineage distribution, and antimicrobial resistance gene (ARG) trends in *S.* Enteritidis strains. (a) Phylogenetic tree constructed from 3255 high-quality *S*. Enteritidis isolates genomes (2322 from China, 933 global). Isolates that have been phylogenetically analyzed are listed in Supplementary Table S2. The long branches leading from the root were truncated and indicated by a ‘||’ symbol to improve visualization of the internal clades. Ring 1: location of each isolate. Ring 2: Bayesian lineage typing. Ring 3: isolation source. (b) Proportion of three major lineages in Chinese Mainland across years. (c) Average number of ARGs carried by strains of different lineages over time. Squares marked with an ‘X’ indicate no strains of that lineage were isolated in that period. (d) Linear relationship between the average number of ARGs and the compound annual growth rate of lineages, with the gray area representing the 95% confidence interval. Pearson correlation analysis shows a significant positive correlation (*P* = 0.01215).

Incorporating 2322 Chinese *S.* Enteritidis genomes with 933 high-quality global genomes (validated via Checkm2; Fig. S1a), we established a maximum-likelihood phylogeny to delineate distinct evolutionary lineages (Table S2). Consistent with established nomenclature, global isolates were classified into the Central/Eastern Africa, West Africa, Outlier, Global (GC-a, GC-b, GC-c), and other predicted clades [1,12]. Our analysis reveals that Chinese isolates predominantly cluster within the Global clades, with only a small fraction belonging to Africa-associated lineages, potentially representing sporadic intercontinental transmission events. Notably, we observed that the Global-b clade has further diverged into two distinct sub-clades in China: GC-b1 and GC-b2. While GC-a exhibited a restricted regional signature, primarily localized to Taiwan province, the majority of isolates from the Chinese Mainland belong to the GC-b1, GC-b2, and GC-c (Fig. 3a).

The temporal dynamics of these three major Chinese Mainland lineages suggest a pattern of successive replacement. GC-c emerged in 2007 and underwent rapid expansion over the last decade. In contrast, GC-b2 circulated primarily between 2012 and 2018 before declining, and GC-b1—though dominant prior to 2006—has shown a steady decrease in prevalence (Fig. 3b). To identify factors linked to this lineage expansion, we quantified ARG burden across clades. GC-c consistently carried the highest number of ARGs compared to GC-b1 and GC-b2 (Fig. 3c). Pearson correlation analysis indicated a significant positive association between the average ARG count and the compound annual growth rate of lineages (*P* = 0.01215, *R*² = 0.1472), suggesting that a higher resistance burden facilitates more rapid expansion. This was further corroborated by a non-parametric Spearman correlation (*ρ* = 0.523, *P* = 0.00038), confirming a monotonic positive trend. While statistically significant, the moderate *R*² value indicates that factors beyond AMR likely contribute to lineage fitness (Fig. 3d and Table S3).

Within GC-c clade, *aph(3'')-Ib, aph(6)-Id, bla*_TEM‑1b_ and *sul2* were enriched (Fig. S2a). MGE profiling further revealed widespread detection of *IncX1* plasmids and the transposons *Tn*6205, *Tn*6292, and *Tn*801, all of which are strongly associated with these determinants (Fig. S2b). Phenotypic MIC assays confirmed that GC-c strains exhibit heightened resistance to aminoglycosides and *β*-lactams—agents commonly used to treat *Salmonella* diarrhea or bacteremia—suggesting that MGE–driven resistance has facilitated clinical escape and thereby promoted the proportional expansion within the Enteritidis lineages (Fig. S3a and Table S4).

Beyond antimicrobial advantages, we evaluated whether the GC-c clade possesses enhanced environmental resilience. Stress response assays (acid, alkaline, oxidative, and thermal) revealed that while GC-c exhibited slightly lower acid tolerance compared to GC-b1 and GC-b2, it showed improved resilience to oxidative and heat stress (Fig. S3b and Table S5). Furthermore, biochemical profiling indicated superior metabolic versatility in GC-c strains; over 50% of GC-c strains demonstrated robust metabolic capabilities across 13 different combinations of oxygen conditions and carbon sources (Fig. S3c and Table S6). No significant differences in biofilm formation were observed between lineages (Fig. S3d and Table S7), suggesting that the evolutionary success of GC-c is primarily driven by a combination of high-level AMR, oxidative/thermal stress tolerance, and metabolic plasticity.

### Genomic signatures of host adaptation and phenotypic divergence in the GC-c lineage

**Figure 4.**
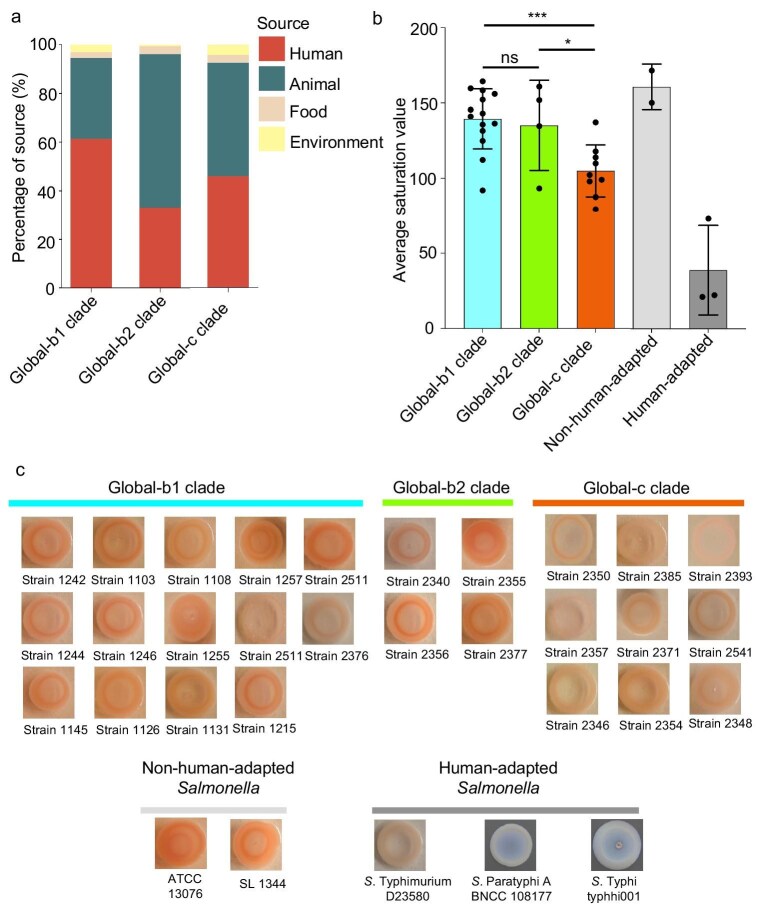
Host composition across lineages and differences in the RDAR phenotypes of the colonies. (a) In different lineages, the proportion of isolates from different sources. (b) Average saturation values of colony color, quantified using ImageJ, for strains across lineages and reference strains. (c) RDAR biofilm formation ability of strains from different lineages on Congo red plates. Five reference strains were used as controls: non-human-adapted strains ATCC 13076 and SL1344; the human-adapted *Salmonella* Typhimurium ST313 strain D23580; *Salmonella* Paratyphi A, and *Salmonella* Typhi.

The prevalence of human clinical isolates within the GC-c lineage (57.2%) was significantly higher than in GC-b1 (31.4%) or GC-b2 (43.2%) (Fig. 4a). Phenotypic characterization revealed that GC-c strains lacked the RDAR (red, dry, and rough) morphotype, exhibiting a colony morphology characteristic of human-adapted *Salmonella* rather than generalist lineages. Quantitative ImageJ analysis of colony saturation confirmed that the phenotypic profile of GC-c differed significantly from both GC-b1 (*P* = 0.0003) and from GC-b2 (*P* = 0.0392) (Fig. 4b and c).

To identify the genetic drivers of this divergence, we compared the GC lineages to the reference strain P125109. We identified a high frequency of non-synonymous mutations across all three clades, with five specific genes in GC-c—*shdA, bapA, cydB, bcsG*, and *focA*—harboring an exceptionally high mutational burden (Fig. S4a and Table S8). Notably, we identified premature stop codons in *shdA* and *bcsG*, resulting in truncated proteins and potential loss of function (Fig. S4b). The truncation of *bcsG*, a gene involved in cellulose biosynthesis, likely underpins the loss of the RDAR morphotype—a hallmark of human adaptation previously documented in invasive *S.* Typhimurium ST313 [28]. Furthermore, *shdA* is a known mediator of intestinal persistence and adhesion in animal hosts [29], suggesting that its pseudogenization may reflect host restriction. Other highly mutated genes in GC-c include *cydB*, which encodes a cytochrome *bd* quinol oxidase subunit involved in oxidative stress defense and resistance to host-derived antimicrobial radicals [30]; *focA*, a formate transporter essential for maintaining acid-base homeostasis and stress adaptation [31]; and *bapA*, which is critical for biofilm formation [32]. Together, these genomic modifications suggest that the expansion of the GC-c lineage is supported by a transition from an environmental/animal-resilient lifestyle to a more specialized, human-adapted pathogenic profile.

### Distinguishable transmission clusters in animals and humans

**Figure 5.**
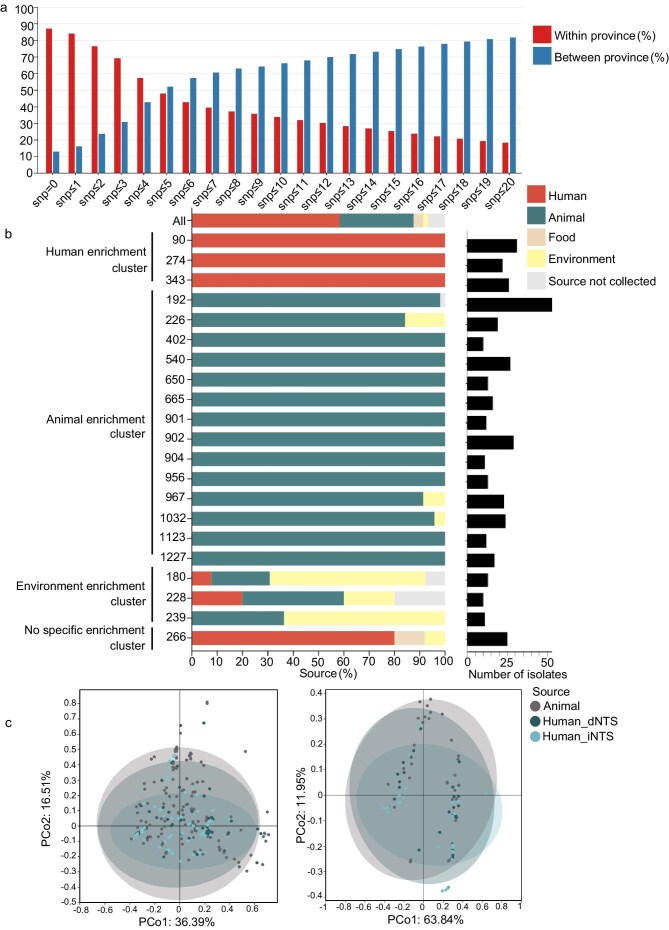
SNP-based transmission analysis; ARGs and MGEs profile. (a) Pairwise SNP distances calculated for strains in this study. Strains from the same province were classified as within-province; those from different provinces were classified as between-province. Proportions of these events were calculated using various SNP distance thresholds (from 0 to 20). (b) Transmission clusters extracted using an SNP distance threshold of ≤4. The left bars show the proportion of strains from different sources in each cluster; the right black bars indicate the number of strains per cluster. The composition ratios of hosts in each cluster were subjected to a Fisher’s exact test across the overall data, and those showing significant differences (*P* < 0.05) were identified as enrichment clusters of the corresponding host origin. The statistical test data are detailed in Supplementary Table S9. (c) Principal component analysis of antimicrobial resistance gene and MGE profiles across isolates from different sources, including animal, human diarrheal isolates (dNTS), and human invasive isolates (iNTS).

To delineate transmission dynamics, we systematically evaluated pairwise SNP distances across the maximum-likelihood phylogeny. By integrating these distances with sampling metadata, we assessed within-province versus between-province transmission events using thresholds ranging from 0 to 20 SNPs. We observed that within-province events predominated when the distance was ≤4 SNPs, a threshold congruent with established criteria for defining probable transmission in *S*. Enteritidis [12] (Fig. 5a). Using this cutoff, we identified ‘Potential Transmission Clusters,’ defined as groups where every member pair falls within the threshold (Table S9). Clusters containing ≥10 isolates were designated as Major Potential Transmission Clusters (MPTCs), totaling 21 distinct clusters.

Fisher’s exact tests were employed to assess host-source enrichment within these MPTCs relative to the overall dataset. Notably, 20 of the 21 major clusters exhibited significant host preference (*P* < 0.05; Table S9), indicating a high degree of host-restricted transmission. This included three specific clusters (Cluster-90, Cluster-274, and Cluster-343), which were almost exclusively associated with human clinical cases, suggesting sustained human-to-human transmission (Fig. 5b). These findings from our nationwide One Health survey underscore that *S*. Enteritidis is increasingly developing host-specific transmission niches within the Chinese epidemiological landscape.

Furthermore, we investigated the distribution of genomic resistance determinants across different host interfaces. Principal component analysis revealed a distinct descending gradient of ARG and MGE abundance (Fig. 5c). The highest richness of resistance determinants was localized in animal-derived isolates, followed by human diarrheal isolates, with the lowest abundance observed in human invasive isolates. This stepwise reduction suggests that while high AMR loads are maintained in the high-pressure environment of the agricultural sector, the transition to invasive systemic infection in humans may involve a selective bottleneck or a fitness-related reduction in the accessory resistome.

## DISCUSSION

This study presents the largest single-nation genomic dataset of *S*. Enteritidis in China, capturing isolates across diverse sources and geographic regions. While Enteritidis has low genetic diversity and is globally dominated by ST11, our extensive sampling revealed notable non-ST11 isolates exhibiting potential host-specific adaptations [33]. Furthermore, we identified a profound geographic dependency in lineage distribution. Most notably, over 85% of isolates from Taiwan province belong to the GC-a lineage, showing a stark contrast to the Chinese Mainland, which is likely driven by geographic isolation and restricted cross-strait exchanges. This contained island environment offers a unique, low-cost model for studying bacterial evolutionary trajectories.

A critical finding of this study is the alarming escalation of MDR among Chinese *S*. Enteritidis, which has surged from 22% to 72% over the past two decades. Surprisingly, environmental isolates displayed the highest prevalence of resistance determinants. This is likely driven by agricultural practices, where persistent exposure to heavy metals and farm disinfectants induces mutations and co-selects for ARGs [27,34,35]. This substantial AMR burden poses a severe challenge to clinical management and underscores the urgency of targeted interventions.

Within the Chinese Mainland, the GC-c lineage has undergone rapid clonal expansion over the past decade. This dominance appears driven by a combination of MGE-mediated antimicrobial resistance and enhanced environmental resilience. Phenotypically, GC-c isolates demonstrate broadened tolerance to environmental stressors, superior metabolic versatility, and a notable regression of the RDAR (red, dry, and rough) morphotype—a trait correlative with human adaptation in invasive African ST313 strains. Genomically, we identified key mutations, including a premature stop codon in *shdA* (often lost in human-adapted *S.* Typhi) and mutations in *bcsG, cydB, focA*, and *bapA* [36]. While these phenotypic and genotypic shifts likely facilitate survival in host environments and during agricultural processing, experimental validation is required to confirm their exact roles in host adaptation. In addition, given that preferences related to host age and sex are reported in *Salmonella* infections, the dynamic interplay between strain evolution and host characteristics should also be considered a key direction for future research [37–39].

Moving beyond traditional similarity analyses, our dataset provides empirical evidence for hidden intra-animal and inter-human transmission networks [40–42]. Notably, Principal Component Analysis of resistance determinants suggests a stepwise transmission model between animal and human populations. As *S.* Enteritidis transitions from the high-selective-pressure environment of livestock farming to causing human invasive disease, there appears to be a progressive loss of resistance determinants. Isolates may shed unnecessary resistance genes associated with agricultural disinfectants, retaining only those necessary to combat commonly used human clinical antimicrobials.

While this study provides a high-resolution framework of *S.* Enteritidis population dynamics, several limitations exist. The uneven representation of host sources (particularly the scarcity of food and environmental isolates) may lead to an underestimation of host-restricted transmission clusters. Additionally, limited historical clinical metadata and geographic sampling imbalances inherent in retrospective genomic studies require that these findings be extrapolated globally with caution. Despite these constraints, this study provides vital insights into lineage-specific host preferences and AMR-driven lineage replacement, underscoring the imperative of sustained, integrated One Health surveillance is imperative to mitigate future pandemic risks.

## CONCLUSION

By applying a comprehensive One Health framework, we have established the largest single-nation genomic dataset of *S*. Enteritidis in China. Our findings highlight a severe and escalating antimicrobial resistance burden that is actively driving lineage replacement and ecological niche adaptation. Furthermore, the identification of host-restricted transmission networks—most notably, genomic evidence of sustained human-to-human transmission—marks a critical shift in the pathogen’s epidemiological profile. Ultimately, this study provides unprecedented insights into the localized evolutionary dynamics of *S*. Enteritidis, offering a vital data-driven foundation for targeted public health interventions and the mitigation of future cross-sectoral transmission risks.

## MATERIALS AND METHODS

### Genomic data collection

The bacterial genomes were obtained from publicly accessible databases and laboratory sources (Fig. S1a and Table S1). For *S*. Enteritidis from China, we retrieved 106 genomes from NCBI, 620 from Enterobase [43], and 1689 from strains routinely monitored by our laboratory. Besides, 936 *S*. Enteritidis genomes from other countries were used to provide global context [1]. Additionally, the isolates were grouped into temporal intervals: ≤2003, 2004–07, 2008–11, 2012–15, 2016–19, and 2020–23—primarily to ensure comparable durations across groups; the detailed distribution of samples by year is shown in Table S1.

### Phylogenetic analysis

Snippy v4.4.4 was used to identify cgSNPs using the complete genome of *S*. Enteritidis P125109 (accession: GCA_000009505.1) as the reference and *S. gallinarum* (accession: GCA_009855385.1) as the outgroup. Genome assemblies of each isolate were aligned to the reference using the Snippy pipeline, which internally employs BWA-MEM for alignment and FreeBayes for variant calling. Only high-quality SNPs with a minimum read coverage of 10× and a minimum variant allele fraction of 0.9 were retained according to the default filtering parameters implemented in Snippy. Core SNPs present in all isolates were extracted using snippy-core. To remove recombination signals, the core SNP alignment was analyzed with Gubbins v2.3 [44], and recombination regions identified by Gubbins were excluded. Only the filtered polymorphic SNP sites were retained for downstream phylogenetic analysis. The recombination-filtered SNP alignment was used to construct a maximum-likelihood phylogenetic tree using IQ-TREE v1.6.12, with automatic model selection (ModelFinder) and 1000 ultrafast bootstrap replicates. The best-fit substitution model selected was TVM + F + ASC + R3. Filtered SNP alignments were then used as input for population structure analysis with RhierBAPS v1.1.4 [45], which implements the Hierarchical Bayesian Clustering algorithm. The SNP alignment in FASTA format generated by Gubbins was used as input. We set a maximum hierarchy depth of 7 and allowed up to 20 initial populations, providing sufficient resolution to capture sub-lineage diversity within the dataset of 3255 genomes while minimizing over-partitioning. No minimum cluster size constraint was imposed, and clusters with small sample sizes were retained to preserve population diversity.

To assess the consistency of the RhierBAPS clustering results, we compared the inferred population structure with the topology of the maximum-likelihood phylogeny. The identified lineages corresponded to well-supported clades in the phylogenetic tree. Additionally, multilocus sequence typing (MLST) information was used as an independent reference. As shown in Table S2, most STs corresponded to unique RhierBAPS lineages (e.g. ST8055 corresponds to the Global-b1 clade and ST1974 corresponds to the Outlier clade), whereas the dominant ST11 lineage was further subdivided into multiple phylogenetic lineages, reflecting its greater genetic diversity.

## Supplementary Material

nwag275_Supplemental_Files

## Data Availability

The accession numbers of all the strains used in this study are provided in Table S1. Assembled Illumina sequence data are available by accession number or from the National Center for Biotechnology Information under the BioProject number: PRJNA1255313. Abricate v1.0.1 (https://github.com/tseemann/abricate), Snippy v4.4.4 (https://github.com/tseemann/snippy), SISTR v1.1.1 (https://github.com/phac-nml/sistr_cmd), SNP-dists v0.7.0 (https://github.com/tseemann/snp-dists), IQtree v1.6.12 (https://github.com/iqtree/iqtree2), Rhierbaps v1.1.4 (https://github.com/gtonkinhill/rhierbaps), Bacant v3.4.0 (https://github.com/xthua/bacant), SPAdes v3.12.0 (https://github.com/ablab/spades), CheckM2 v1.1.0 (https://github.com/chklovski/CheckM2), KmerFinder v3.2 (https://github.com/CaileanCarter/kmerFinder), MLST v2.22.0 (https://github.com/tseemann/mlst), Resfinder (https://github.com/cadms/resfinder), PlasmidFinder (https://github.com/genomicepidemiology/plasmidfinder) INTEGRALL (http://integrall.bio.ua.pt), The Transposon Registry (https://transposon.lstmed.ac.uk/tn-registry), Prokka v1.14.6 (https://github.com/tseemann/prokka), Roary v3.13.0 (https://github.com/sanger-pathogens/Roary), Gubbins v2.3 (https://github.com/nickjcroucher/gubbins).
