# Supplementary material for: One Health genomics reveals niche-specific lineage replacement in Salmonella Enteritidis
Source: Natl Sci Rev. 2026 May 12;13(11):nwag275. doi: 10.1093/nsr/nwag275 (PMC13281096; doi:10.1093/nsr/nwag275)
Supplement: nwag275_Supplemental_Files [file nwag275_supplemental_files.zip › Supplementary Figure 1_170x100_260422.pdf]

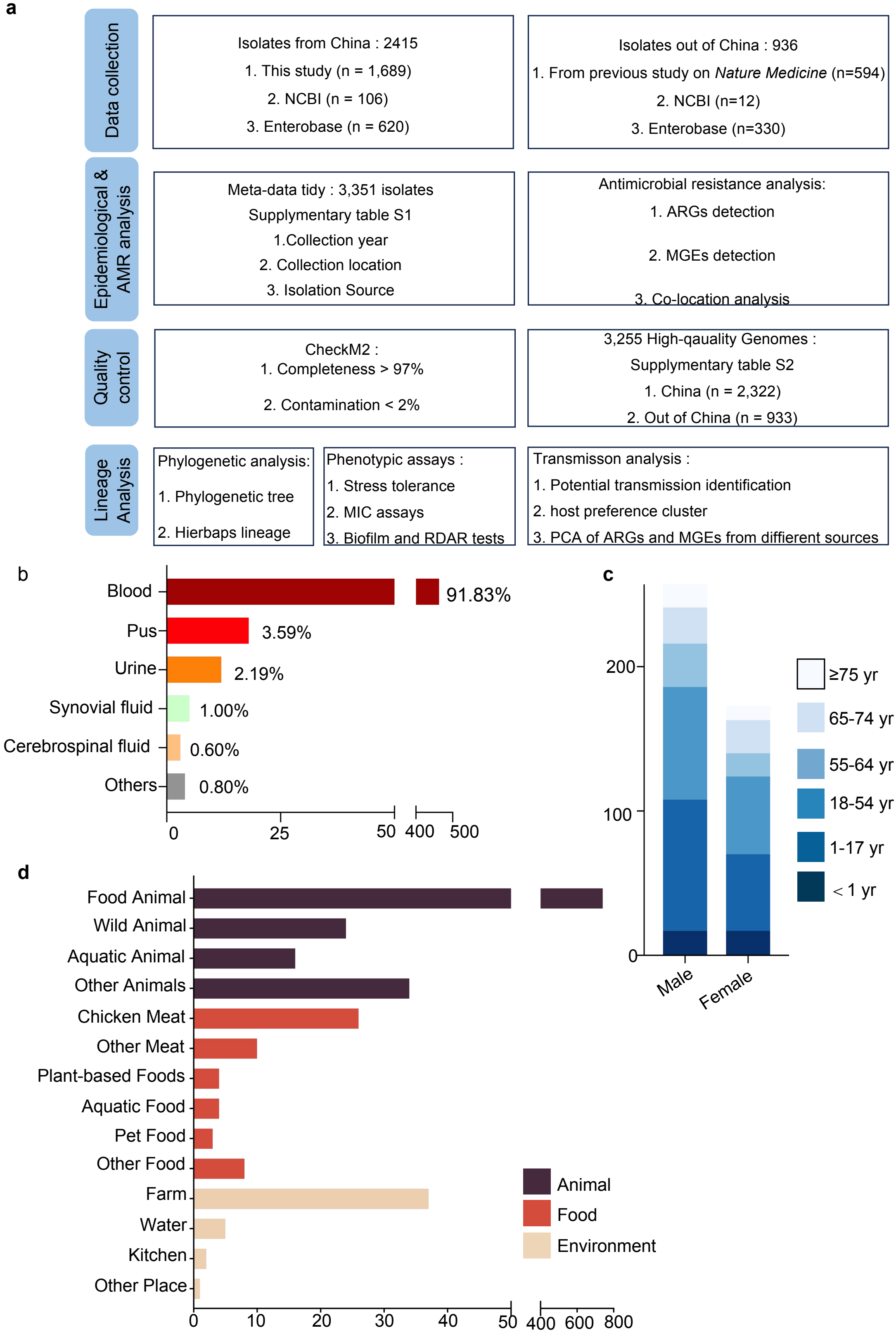
a
Data collection
Isolates from China : 2415
1. This study (n = 1,689)
2. NCBI (n = 106)
3. Enterobase (n = 620)
Isolates out of China : 936
1. From previous study on Nature Medicine (n=594)
2. NCBI (n=12)
3. Enterobase (n=330)
Epidemiological & AMR analysis
Meta-data tidy : 3,351 isolates
Supplymentary table S1
1.Collection year
2. Collection location
3. Isolation Source
Antimicrobial resistance analysis:
1. ARGs detection
2. MGEs detection
3. Co-location analysis
Quality control
CheckM2 :
1. Completeness > 97%
2. Contamination < 2%
3,255 High-qauality Genomes :
Supplymentary table S2
1. China (n = 2,322)
2. Out of China (n = 933)
Lineage Analysis
Phylogenetic analysis:
1. Phylogenetic tree
2. Hierbaps lineage
Phenotypic assays :
1. Stress tolerance
2. MIC assays
3. Biofilm and RDAR tests
Transmisson analysis :
1. Potential transmission identification
2. host preference cluster
3. PCA of ARGs and MGEs from diffierent sources
b
Blood
Pus
Urine
Synovial fluid
Cerebrospinal fluid
Others
91.83%
3.59%
2.19%
1.00%
0.60%
0.80%
0
25
50
400
500
c
0
100
200
Male
Female
≥75 yr
65-74 yr
55-64 yr
18-54 yr
1-17 yr
< 1 yr
d
Food Animal
Wild Animal
Aquatic Animal
Other Animals
Chicken Meat
Other Meat
Plant-based Foods
Aquatic Food
Pet Food
Other Food
Farm
Water
Kitchen
Other Place
Animal
Food
Environment
0
10
20
30
40
50
400
600
800
