# Supplementary material for: One Health genomics reveals niche-specific lineage replacement in Salmonella Enteritidis
Source: Natl Sci Rev. 2026 May 12;13(11):nwag275. doi: 10.1093/nsr/nwag275 (PMC13281096; doi:10.1093/nsr/nwag275)
Supplement: nwag275_Supplemental_Files [file nwag275_supplemental_files.zip › Supplemetary data_260422.docx]

**One Health Genomics Reveals Niche-Specific Lineage Replacement in *Salmonella* Enteritidis**

Haiyang Zhou^1,2 #^, Linlin Huang^1 #^, Jiansen Gong^3 #^, Chenghao Jia^1,2^, Qianzhe Cao^1^, Zubin Wang^2^, Yan Song^1^, Jiaqi Chen^1^, Aizhong Chen^2^, Yi Zhang^2^, Abdelaziz Ed-Dra^4^, Yan Li^1^, Guoping Zhao^2,5,6^, Min Yue^1,2,7^ *

**Affiliations**

1 Department of Veterinary Medicine, Zhejiang University College of Animal Sciences, Hangzhou 310058, China

2 Key Laboratory of Systems Health Science of Zhejiang Province, School of Life Science, Hangzhou Institute for Advanced Study, University of Chinese Academy of Sciences, Hangzhou 310024, People’s Republic of China

3 Key Laboratory for Poultry Genetics and Breeding of Jiangsu Province, Jiangsu Institute of Poultry Sciences, Yangzhou 225125, China

4 Laboratory of Engineering and Applied Technologies, Higher School of Technology, M’ghila Campus, Sultan Moulay Slimane University, BP: 591, Beni Mellal 23000, Morocco.

5 CAS Key Laboratory of Synthetic Biology, Institute of Plant Physiology and Ecology, Shanghai Institutes for Biological Sciences, Chinese Academy of Sciences, Shanghai 200031, China

6 Department of Microbiology and Microbial Engineering, School of Life Sciences, Fudan University, Shanghai 200433, China

7 State Key Laboratory for Diagnosis and Treatment of Infectious Diseases, National Clinical Research Center for Infectious Diseases, National Medical Center for Infectious Diseases, The First Affiliated Hospital, College of Medicine, Zhejiang University, Hangzhou 310003, China.

# These authors contributed equally to this work

* Corresponding author:

Min Yue. Tel: +86 0571 88982832; E-mail: myue@zju.edu.cn

**SUPPLEMENTARY MATERIALS AND METHODS**

***S*. Enteritidis isolation and identification**

The isolation and identification of *S*. Enteritidis were carried out according to the methods described in our previous work[1]. Tetrathionate Broth Base (TTB, Land Bridge Biotechnology Co, Beijing, China) and Xylose Lysine Deoxycholate (XLD, Land Bridge Technology Co, Beijing, China) agar were used to select *Salmonella* colonies. Typical and pure colonies were selected after sub-culturing on XLD agar and transferred to Luria-Bertani (LB) broth. The bacterial cultures were then further incubated at 37°C for 18-22 hours in a rotary incubator set to 180 rpm. *S.* Enteritidis isolates were confirmed using a PCR assay and serotyping based on the Kauffman white scheme as documented previously [2,3].

**DNA extraction, genomic sequencing and assembly**

For the collection of *S*. Enteritidis isolates provided by our laboratory, DNA was extracted using the Vazyme Fastpure® Bacteria DNA Isolation Mini Kit (Vazyme Biotech Co., Ltd.) and quantified with the NanoDrop1000 system (Thermo Fisher Scientific, USA). DNA libraries were constructed and sequenced on the Illumina Novaseq 6000 platform (Novogene, Beijing, China). Genome sequences were assembled using SPAdes v3.12.0 with default settings[4]. CheckM2 v1.1.0 was employed to assess genome quality[5] (**Supplementary Table S2**). 3,255 genomes with >97% completeness and <2% contamination are kept for downstream phylogenetic analyses[6]. The bacterial species and biovar type associated with each genome were confirmed using KmerFinder v3.2.

**Biofilm formation, Red, dry, and rough (RDAR) phenotype of colony, and Tolerance Assay**

Overnight bacterial cultures were diluted 1:1000 in LB broth and inoculated into flat-bottom 96-well microtiter plates (200 µL/well), with *E. coli* ATCC 25922 as a positive control and uninoculated LB broth as a negative control. Five replicates per strain were tested at 37°C and 28°C for 5 days (static incubation). Edge wells were filled with sterile water to minimize evaporation. Post-incubation, planktonic cells were removed by washing (3× with distilled water), and adherent biofilms were fixed (60°C, 30 min), stained with 0.4% crystal violet (25 min), washed, and solubilized with 75% ethanol. OD_550nm_ was measured using a Tecan spectrophotometer, with biofilm formation quantified by subtracting blank OD values. 2μL of bacterial culture (OD_600_ 1.0) was spotted onto Congo Red-TB agar. After static incubation at 28°C for 48–72 h, colony morphology and red pigmentation were assessed visually or using ImageJ, as described previously [7].

Overnight cultures were diluted 1:100 in fresh LB broth and incubated at 37°C with shaking (220 rpm) until an OD_600_ of 0.4–0.5 was reached. Initial CFU counts were obtained by spot-plating on LB agar. Bacterial cells were diluted in 0.9% NaCl and treated under different conditions (pH 3.0 with HCl, pH 10.0 with NaOH, or 10 mM H_2_O_2_), incubated statically at 37°C for 30 minutes, and then plated on LB agar. Survival rates were determined as log_10_ (CFU post-treatment / initial CFU). For heat-stress experiments, overnight cultures at OD_600_ of 0.4–0.5 were diluted into 0.9% NaCl and incubated at 42°C for 10 minutes.

**Biochemical assays**

*S*. Enteritidis isolates were streaked onto an antibiotic-free LB plate for recovery and incubated at 37°C for 18–24 hours. A single colony was selected and transferred to antibiotic-free LB liquid medium, cultured at 37°C with shaking (180 r/min) for 18–24 hours. The bacterial solution was washed with PBS, and the optical density (OD_600_) was adjusted to approximately 0.1. Then, 30 μL of the bacterial solution was added to each biochemical reaction tube, which were incubated under aerobic and anaerobic conditions for 18–24 hours. Color changes and gas production were observed, and results were recorded. A control tube without bacteria was included, and three replicates were performed for each strain and reaction. The biochemical metabolism tests include mannose, D-xylose, trehalose, L-arabinose, L-tartaric acid, Simmons citrate, mucate, dulcitol, H2S, L-rhamnose, and maltose.

**Antimicrobial susceptibility testing**

The minimum inhibitory concentration (MIC) of 13 clinically relevant antimicrobials was determined by broth microdilution method, following Clinical and Laboratory Standards Institute (CLSI) guidelines. *Escherichia coli* ATCC 25922 served as the control strain. The tested antimicrobials and their concentration ranges (mg/L) included: penicillins (ampicillin: AMP, 0.5–64), β-lactams (amoxicillin-clavulanic acid: AMO, 0.25–128), aminoglycosides (gentamicin: GEN, 0.25–32; kanamycin: KAN, 0.5–64; streptomycin: STR, 0.5–64), tetracyclines (tetracycline: TET, 0.5–64), quinolones (ciprofloxacin: CIP, 0.06–16; nalidixic acid: NAL, 0.5–64), folate pathway inhibitors (trimethoprim-sulfamethoxazole: SXT, 8–1,024), macrolides (azithromycin: AZM, 0.5–64), phenicols (chloramphenicol: CHL, 0.5–64), and cephems (ceftriaxone: CRO, 0.5–64; ceftiofur: CX, 1–128).

**Bioinformatics analysis**

All genome sequences used in this study were analyzed for serovar prediction using SISTR v1.1[8]. Initial typing of isolates was done via MLST (Galaxy Version 2.22.0)[9]. Antimicrobial resistance genes (ARGs) were identified using ABRicate v1.0.1 with the ResFinder database (downloaded via ABRicate on 2025-02-20, minimum identity=90.0, minimum coverage=90.0)[10]. Plasmids were detected according to Plasmidfinder (downloaded via ABRicate on 2025-02-20, minimum identity=95.0, minimum coverage=90.0)[11].

The BacAnt v3.4.0 tool was employed to identify integrons and transposons within the genomes of *Salmonella*, as we described before[12]. It utilized NCBI, INTEGRALL, and THE TRANSPOSON REGISTRY databases, detecting only those integrons or transposons with over 60% similarity and 60% coverage[13].

**Visualization and Statistical Analysis**

GraphPad Prism 8.0 (San Diego, CA, United States) and Python v3.9.13 were used for data analysis and figure generation. The chi-square test, t-test, and Fisher's exact test were used to assess significant differences between groups, as well as the differences in the carrier rates of related genes across lineages. A *p*-value of less than 0.05 was considered statistically significant.

**SUPPLEMENTARY FIGURE LEGENDS**

**Supplementary Figure 1. Genomic Data Collection, Quality Control, and Characteristics of Bacterial Strains.**

a. Workflow for data collection, quality control, and analysis process in this study.

b. Specific isolation sites of iNTS strains, predominantly from blood, followed by pus, urine, cerebrospinal fluid, synovial fluid, and others.

c. Age and gender distribution of iNTS patients, categorized by age groups: ≥75 years, 65–74 years, 55–64 years, 18–54 years, 1–17 years, and <1 year.

d. Detailed distribution of animal, food, and environmental sources.

**Supplementary Figure 2. Antimicrobial Resistance Genes, Mobile Genetic Elements Across Lineages.**

a. Detection of specific ARGs across different lineages. The gene *aac(6')-Iaa* was excluded from the analysis. The intensity of red indicates the percentage of strains carrying each resistance gene.

b. Detection of mobile genetic elements highly associated with resistance genes across lineages. The intensity of red reflects the percentage of strains carrying each mobile element.

**Supplementary Figure 3. Stress Tolerance assays, Metabolic Capabilities, Biofilm Formation, and AMR profile Across Lineages.**

a. ARGs-based genotype and MIC-based phenotype of resistance rates to 11 antimicrobial classes and specific drugs (Kanamycin, KAN; Gentamicin, GEN; Streptomycin, STR; Penicillins, AMP; Amoxicillin-clavulanic acid, AMO; Ceftiofur, CX; Ceftriaxone, CRO; Nalidixic acid, NAL; Ciprofloxacin, CIP; Chloramphenicol, CHL; Azithromycin, AZM; Tetracycline, TET; Trimethoprim-sulfamethoxazole, SXT).

b. Stress resistance of Global-b1 Clade, Global-b2 Clade and Global-c Clade under four conditions (42°C, H₂O₂, pH 3, pH 10), assessed by the log_10_ survival ratio of strains post-treatment. The list of isolates has been examined, and the results are provided in Supplementary Table S5. In the box-and-whisker plots, the box represents the interquartile range (IQR), with the lower and upper boundaries corresponding to the 25th percentile (Q1) and 75th percentile (Q3), respectively. The horizontal line within the box indicates the median.

c. Biochemical metabolism of 11 substrates under aerobic (the first row) and anaerobic (the second row) conditions by different lineages (Global-b1 Clade, Global-b2 Clade, Global-c Clade, and overall), with percentages indicating the proportion of strains showing a positive reaction. Color intensity is reported as a percentage, with two replicates per substrate. The list of isolates has been tested, and the results are provided in Supplementary Table S6.

d. Biofilm formation capacity of different lineages (Global-b1 Clade, Global-b2 Clade, and Global-c Clade) at 28°C and 37°C, measured by crystal violet staining and absorbance at 550 nm after ethanol elution. Strains ATCC 25922 and mock control are included for comparison. The list of isolates has been tested, and the results are provided in Supplementary Table S7.

**Supplementary Figure 4. Genomic variations and mutations across different lineages.**

a. 5 key mutations in the three major sub-lineages prevalent in Chinese mainland were identified using Snippy, with strain P125109 as the reference. The location of the genetic mutation and the amino acid change are indicated below.

b. Premature stop codons were frequently observed in the *bcsG* and *shdA* genes among the Global-c Clade. A schematic representation of the stop codon positions is provided.

**REFERENCES**

1. Li Y, Kang X, Ed-Dra A, et al. Genome-Based Assessment of Antimicrobial Resistance and Virulence Potential of Isolates of Non-Pullorum/Gallinarum Salmonella Serovars Recovered from Dead Poultry in China. *Microbiol Spectr*. 2022;10(4):e0096522. doi:10.1128/spectrum.00965-22

2. Liu Y, Jiang J, Ed-Dra A, et al. Prevalence and genomic investigation of Salmonella isolates recovered from animal food-chain in Xinjiang, China. *Food Res Int*. 2021;142:110198. doi:10.1016/j.foodres.2021.110198

3. Wang H, Kang X, Yu L, et al. Developing a novel TaqMan qPCR assay for optimizing Salmonella Pullorum detection in chickens. *Vet Q*. 2025;45(1)doi:10.1080/01652176.2025.2454473

4. Prjibelski A, Antipov D, Meleshko D, Lapidus A, Korobeynikov A. Using SPAdes De Novo Assembler. *Curr Protoc Bioinformatics*. 2020;70(1):e102. doi:10.1002/cpbi.102

5. Chklovski A, Parks DH, Woodcroft BJ, Tyson GW. CheckM2: a rapid, scalable and accurate tool for assessing microbial genome quality using machine learning. *Nat Methods*. 2023;20(8):1203-1212. doi:10.1038/s41592-023-01940-w

6. Xu X, Lin Y, Deng Y, et al. Ecological connectivity of genomic markers of antimicrobial resistance in Escherichia coli in Hong Kong. *Nat Commun*. 2025;16(1):7319. doi:10.1038/s41467-025-62455-w

7. Wang Z, Jiang Z, Cao Q, et al. A genomic and phenotypic investigation of pigeon-adaptive Salmonella. *PLoS Pathog*. 2025;21(3):e1012992. doi:10.1371/journal.ppat.1012992

8. Yoshida CE, Kruczkiewicz P, Laing CR, et al. The Salmonella In Silico Typing Resource (SISTR): An Open Web-Accessible Tool for Rapidly Typing and Subtyping Draft Salmonella Genome Assemblies. *PLoS One*. 2016;11(1):e0147101. doi:10.1371/journal.pone.0147101

9. Jolley KA, Bray JE, Maiden MCJ. Open-access bacterial population genomics: BIGSdb software, the PubMLST.org website and their applications. *Wellcome Open Res*. 2018;3:124. doi:10.12688/wellcomeopenres.14826.1

10. Bortolaia V, Kaas RS, Ruppe E, et al. ResFinder 4.0 for predictions of phenotypes from genotypes. *J Antimicrob Chemother*. 2020;75(12):3491-3500. doi:10.1093/jac/dkaa345

11. Carattoli A, Zankari E, García-Fernández A, et al. In silico detection and typing of plasmids using PlasmidFinder and plasmid multilocus sequence typing. *Antimicrob Agents Chemother*. 2014;58(7):3895-3903. doi:10.1128/AAC.02412-14

12. Jia C, Wang Z, Huang C, et al. Mobilome-driven partitions of the resistome in Salmonella. *mSystems*. 2023;8(6):e0088323. doi:10.1128/msystems.00883-23

13. Moura A, Soares M, Pereira C, Leitão N, Henriques I, Correia A. INTEGRALL: a database and search engine for integrons, integrases and gene cassettes. *Bioinformatics*. 2009;25(8):1096-1098. doi:10.1093/bioinformatics/btp105
