# Supplementary figures and images for: One Health genomics reveals niche-specific lineage replacement in Salmonella Enteritidis
Source: Natl Sci Rev. 2026 May 12;13(11):nwag275. doi: 10.1093/nsr/nwag275 (PMC13281096; doi:10.1093/nsr/nwag275)

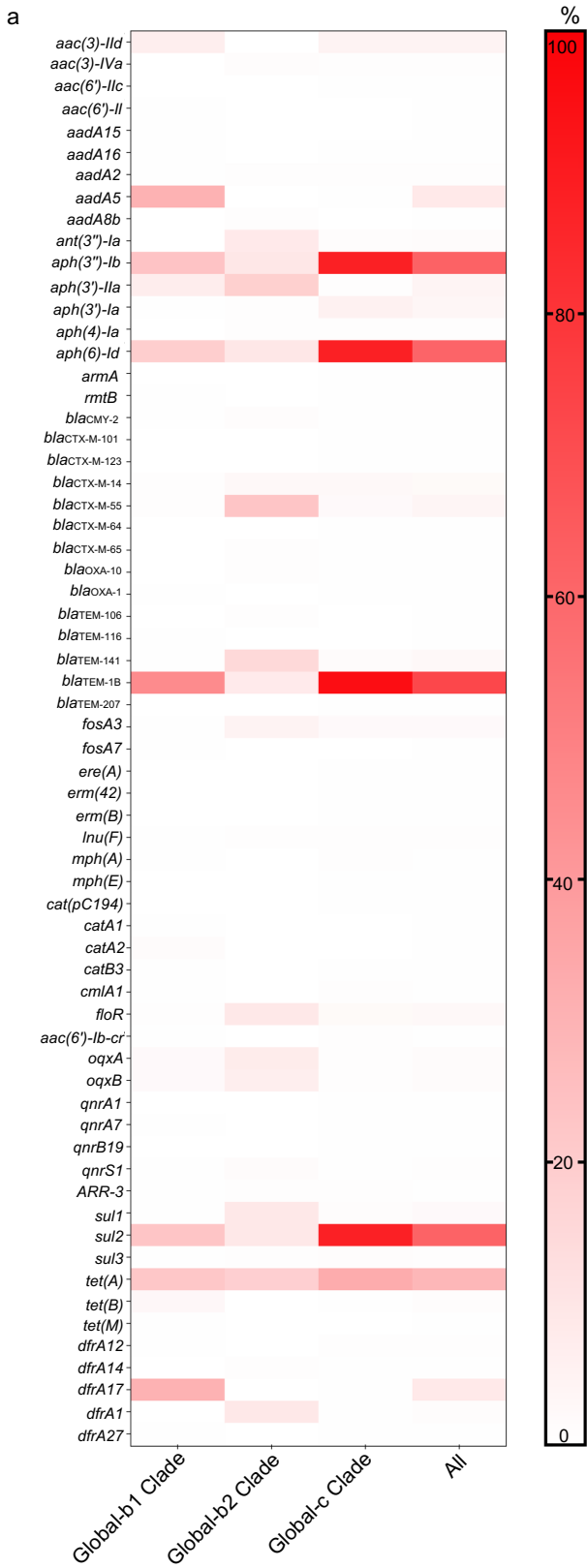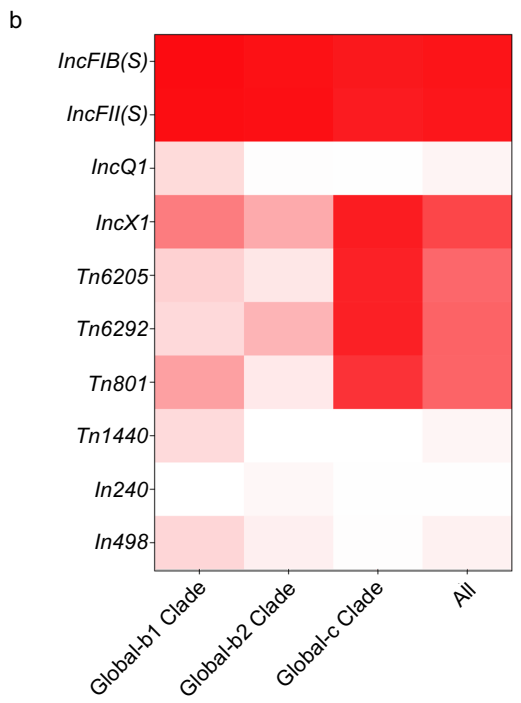

Supplement: nwag275_Supplemental_Files [file nwag275_supplemental_files.zip › Supplementary Figure 2_170x100_260422.pdf]

a

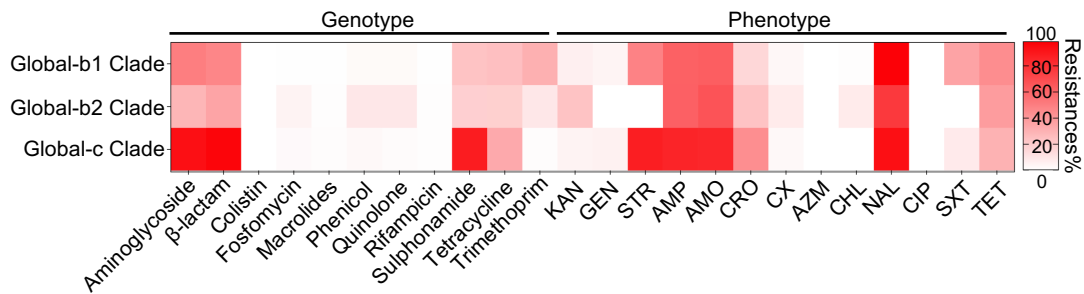

b

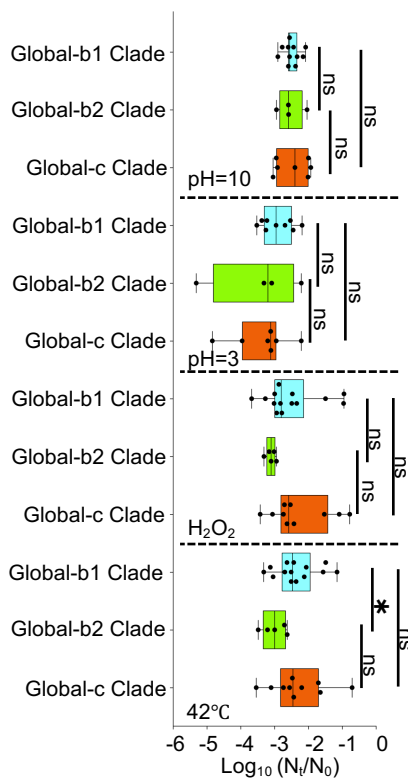

c

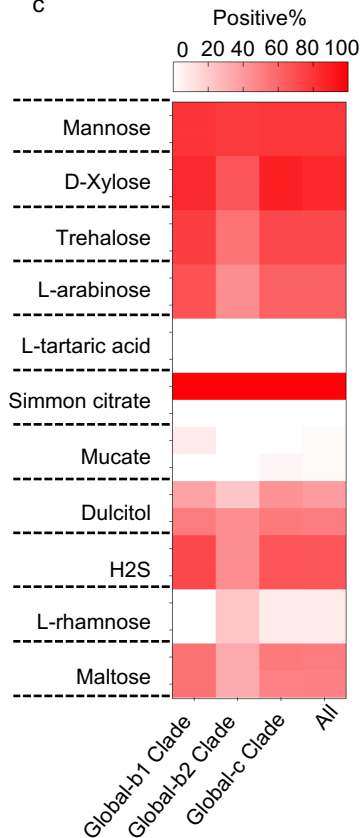

d

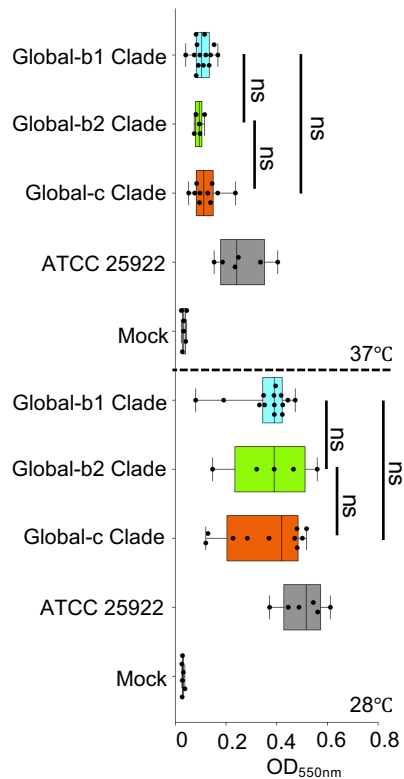

Supplement: nwag275_Supplemental_Files [file nwag275_supplemental_files.zip › Supplementary Figure 3_170x170_260422.pdf.pdf]

a

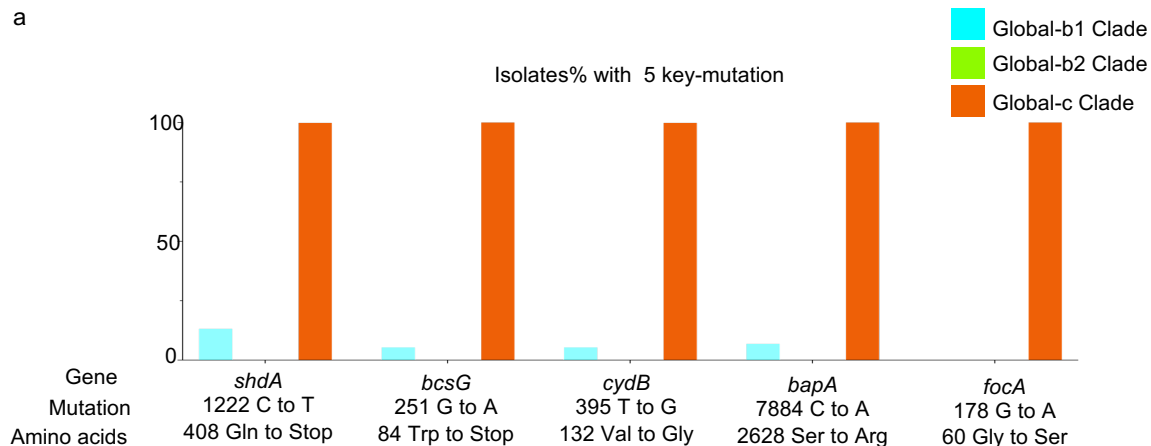

b

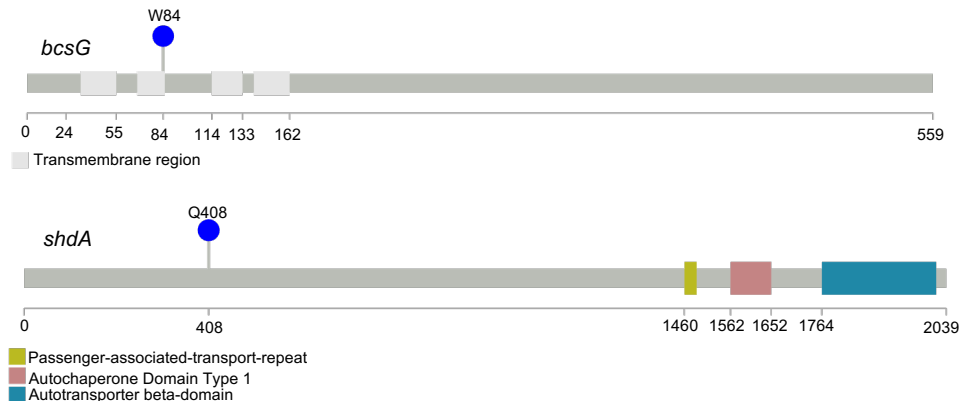

Supplement: nwag275_Supplemental_Files [file nwag275_supplemental_files.zip › Supplementary Figure 4_170x130_260422.pdf.pdf]
